# CuFormer U-Net: a transformer-enhanced multi-task network for cucumber leaf disease detection and segmentation

**DOI:** 10.3389/fpls.2026.1975030

**Published:** 2026-10-07

**Authors:** Batyrkhan Omarov, Sayat Ibrayev, Nurbibi Imanbayeva

**Affiliations:** 1 Laboratory of Mechanics of Robots and Manipulators, Joldasbekov Institute of Mechanics and Engineering, Almaty, Kazakhstan; 2 Department of Mathematical and Computer Modeling, International Information Technology University, Almaty, Kazakhstan

**Keywords:** boundary refinement, cucumber leaf disease, multi-task learning, plant disease, precision agriculture, semantic segmentation, vision transformer

## Abstract

Cucumber foliar symptoms reduce functional leaf area and complicate greenhouse scouting. This study presents CuFormer U-Net, a Transformer-enhanced multi-task network for three-class image-level classification on the 3,754-image Kaggle benchmark (healthy, powdery mildew, and downy mildew) and binary visible-symptom segmentation on a separate 400-image greenhouse cohort (200 healthy and 200 affected leaves). The greenhouse annotations comprise 2,705 rectangles (1,741 color-abnormality and 964 tissue-damage instances); rectangle-seeded lesion masks were manually refined for the dense-prediction experiments. The reported fixed-split point estimates were 96.7% accuracy, 96.3% macro precision, 95.8% macro recall, and 96.0% macro F1 for classification, and 92.4% mean image-wise Dice, 83.1% macro IoU, 93.2% foreground precision, and 91.7% foreground recall for segmentation. The complete internal-validation ablation configuration reached 93.1% Dice and 87.3% IoU. The available experiment archive does not contain independent-seed reruns, per-image predictions for inferential testing, annotator-level masks, quantitative results for the separate 100-image greenhouse stress-test cohort, or reproducible hardware profiling. Accordingly, all between-model differences are interpreted descriptively, the external cohort is not presented as formal external validation, and robustness and deployment claims have been moderated. CuFormer U-Net provides a technically motivated basis for joint recognition and spatial characterization, but confirmatory multi-run and multi-site evaluation is required.

boundary refinement, cucumber leaf disease, multi-task learning, plant disease, precision agriculture, semantic segmentation, vision transformer

## 1 Introduction

### 1.1 Background and motivation

Cucumber (*Cucumis sativus* L.) is a high-value vegetable cultivated in open fields and protected environments because of its short production cycle, dietary importance, and continuous market demand. Intensive greenhouse cultivation increases yield per unit area, but it also creates humid microclimates that favor downy mildew, powdery mildew, angular leaf spot, anthracnose, and other foliar disorders. Disease symptoms reduce functional leaf area, disturb photosynthesis, accelerate senescence, and can propagate rapidly across spatially dense plant rows. Early diagnosis is therefore essential for preserving yield and limiting unnecessary broad-spectrum pesticide use.

Conventional scouting depends on grower experience and visual inspection, which may confuse disease symptoms with nutrient stress, senescence, glare, or physical damage. A recent review found that variation in illumination, background composition, disease stage, and imaging device remains a central barrier to reliable plant-disease recognition outside controlled datasets (Upadhyay et al., 2025). An image-based review likewise emphasized that deployment reliability requires models to distinguish subtle lesions from natural leaf texture rather than merely exploit dataset-specific backgrounds (Bagga and Goyal, 2024). Computer vision can support repeatable, non-destructive monitoring by converting RGB imagery into class probabilities, lesion masks, severity estimates, and actionable spatial maps.

Deep CNNs have substantially improved agricultural image analysis because hierarchical filters learn color, edge, texture, and shape representations directly from data. Comparative experiments have nevertheless shown that performance changes markedly across ResNet, VGG, DenseNet, and lightweight backbones when image quality and class balance vary (Dahiya et al., 2022). Vision Transformers provide a complementary mechanism by capturing long-range relations among spatial regions. Plant-disease experiments based on Vision Transformers reported strong classification performance but also confirmed sensitivity to dataset size and training protocol (Borhani et al., 2022). Hybrid local-global models are consequently attractive for greenhouse imagery, where small lesions require high-frequency convolutional detail and distributed discoloration requires global contextual reasoning.

### 1.2 Research gap

Five methodological limitations remain prominent. First, CNN receptive fields grow gradually, so purely convolutional networks may represent neighboring lesions accurately while failing to relate spatially separated symptoms across an entire leaf. Second, global self-attention is computationally expensive at high resolution, which restricts direct Transformer processing of 512 × 512 agricultural images. Third, most cucumber studies optimize either image-level classification or lesion localization, preventing explicit interaction between diagnostic identity and spatial extent. Fourth, conventional U-Net skip connections pass all encoder activations to the decoder, including veins, stems, irrigation pipes, specular highlights, and background foliage. Fifth, coarse decoder masks frequently smooth irregular lesion boundaries or merge adjacent spots under occlusion and uneven lighting.

Improved Swin Transformer models have demonstrated the value of hierarchical global context for practical cucumber recognition (Wang et al., 2022). Lightweight YOLO variants have improved small-symptom detection, yet bounding boxes do not quantify affected tissue precisely (Omer et al., 2024). Recent cucumber severity work adopted efficient semantic segmentation to estimate diseased area, but classification and segmentation were not fully optimized as mutually guiding tasks (Li et al., 2025). These observations motivate a unified architecture that restricts expensive attention to a compressed bottleneck, injects disease evidence into dense prediction, filters skip information, and restores lesion boundaries.

### 1.3 Main contributions

The main contributions are as follows:

1. A novel CuFormer U-Net architecture jointly optimizes cucumber disease classification and lesion segmentation through a shared hierarchical encoder and task-specific prediction heads.
2. A multi-scale context module aggregates dilated local evidence and image-level context before a compact Transformer bottleneck models long-range lesion dependencies.
3. Disease-guided feature modulation converts classification probabilities into a spatial conditioning signal that aligns the decoder with diagnostically relevant patterns.
4. Attention-guided skip connections suppress irrelevant greenhouse features by combining encoder detail, decoder context, and the disease embedding.
5. A boundary-aware refinement branch and deep supervision improve delineation of small, fragmented, and irregular lesions.
6. The architectural novelty lies in the directed interaction among modules: global context is restricted to a compact 64-token bottleneck; classification probabilities are projected into a disease embedding; that embedding modulates bottleneck features and conditions each skip gate; and a separate boundary objective refines the final mask. This is distinct from a standard U-Net with independent classification and segmentation heads.

## 2 Related works

### 2.1 Deep learning for plant disease classification

AlexNet and VGG established sequential convolutional feature hierarchies, but their dense layers and repeated convolutions require considerable memory. Agricultural studies retain them as interpretable transfer-learning baselines because their texture filters distinguish high-contrast symptoms. VGG16 exceeded a custom CNN and InceptionV3 on a three-class cucumber dataset, confirming the value of transferred features with limited data (Shahid and Zohaib, 2025). ResNet uses identity shortcuts to stabilize depth, whereas DenseNet promotes feature reuse through dense connectivity. CNN ensembles can increase robustness but multiply inference cost (Shovon et al., 2023).

EfficientNet reduces cost through compound scaling, while MobileNet uses depthwise convolution and inverted residual blocks. Lightweight spatial attention can preserve plant-disease discrimination with fewer parameters (Batool et al., 2025). ConvNeXt modernized CNNs through large kernels, stage-wise scaling, and Transformer-inspired training while retaining convolutional bias (Liu et al., 2022a). ConvNeXt V2 added masked autoencoding and global response normalization (Woo et al., 2023).

Vision Transformer models global token relations but requires strong regularization and sufficient data. Swin Transformer builds a hierarchy through shifted-window attention, and its second version improves high-resolution stability (Liu et al., 2022b). MaxViT alternates blocked and grid attention for efficient local-global interaction (Tu et al., 2022). These models represent scattered symptoms effectively, although pretraining dependence and latency remain concerns. Mixture-of-experts routing can improve wild-condition plant classification at additional system cost (Salman et al., 2025).

### 2.2 Semantic segmentation of plant diseases

FCN replaces dense classifiers with spatial prediction but often yields coarse boundaries after downsampling. SegNet reuses pooling indices, whereas U-Net restores detail through encoder-decoder concatenation. U-Net++ uses nested skips, and Attention U-Net gates encoder features. DeepLabV3+ combines atrous context with a shallow decoder, while PSPNet pools several spatial scales. HRNet maintains parallel high-resolution streams. Mask R-CNN predicts boxes and instance masks, adding complexity when lesions are treated as one semantic class.

Agricultural segmentation increasingly targets severity rather than disease presence alone. Pixel supervision can jointly support classification and damage quantification (Polly and Devi, 2024). Farooqui et al. (2025) showed that nested U-Net-style pathways can support plant-leaf segmentation under limited-sample conditions, reinforcing the need to preserve multi-scale spatial detail. DM-BiSeNet reduced cucumber severity-estimation cost through separate spatial and context pathways (Li et al., 2025). Fine boundaries nevertheless remain difficult when symptoms resemble veins, shadows, or senescence.

### 2.3 Transformer-based medical and agricultural segmentation networks

Medical segmentation provides relevant Transformer evidence because anatomical structures and plant lesions share multi-scale boundary challenges. UNETR combines Transformer encoding with U-shaped decoding for global dependency modeling (Hatamizadeh et al., 2022). UCTransNet demonstrated that selective channel-wise skip interaction can outperform direct concatenation (Wang et al., 2022a). Mask2Former introduced masked cross-attention for universal segmentation (Cheng et al., 2022). SegNeXt achieved broad context with large-kernel convolutional attention rather than full quadratic attention (Guo et al., 2022).

TransUNet places a Transformer at a CNN bottleneck, Swin-Unet uses shifted-window encoding and decoding, and SegFormer couples a hierarchical Transformer with an MLP decoder. EfficientViT provides lightweight multi-scale attention for high-resolution prediction (Cai et al., 2023). Deformable large-kernel attention adapts receptive fields to boundary geometry (Azad et al., 2024). U-MixFormer further balances U-shaped detail recovery and mixed attention (Yeom and von Klitzing, 2025). These advances favor bottleneck-level attention when resolution is high and labels are limited.

### 2.4 Multi-task learning in computer vision

Multi-task learning transfers information among related outputs through shared representations. Shared encoders can reduce computation and improve generalization when task gradients are balanced (Vandenhende et al., 2022). Hard sharing, soft sharing, auxiliary supervision, and task-specific optimization present different negative-transfer risks (Zhang and Yang, 2022). InvPT models spatial and task interaction with an inverted-pyramid Transformer (Ye and Xu, 2022). TaskPrompter instead learns shared and task-specific cues through spatial-channel prompts (Ye and Xu, 2023).

In agricultural vision, classification provides disease identity while segmentation provides spatial evidence and severity. Joint training can regularize the encoder and discourage predictions unsupported by lesions. Uncontrolled sharing may still conflict because classification emphasizes global semantics and segmentation emphasizes boundaries; disease-conditioned modulation offers more structured interaction.

### 2.5 Existing studies on cucumber leaf disease analysis

Recent cucumber studies cover classification, detection, and severity. Multi-level entropy selection fused pretrained deep features (Khan et al., 2022). A tuned CNN reported strong multi-class recognition on an expanded cucumber collection (Omer et al., 2022). Concatenated handcrafted and deep features with a modified LSTM further demonstrated the value of combining complementary crop-disease representations (Farooqui et al., 2023). Image-text-label learning aligned visual and semantic spaces for small-sample recognition (Cao et al., 2023). Flooding-based MobileNet targeted lightweight natural-scene recognition (Liu et al., 2023). DM-YOLO addressed symptom diversity and environmental interference (Ding et al., 2024).

Fuzzy deep learning with chaotic particle-swarm selection provided classification and robustness analysis on five diseases (Bilal et al., 2025). YOLO-Cucumber combined deformable convolution, fine-resolution prediction, target-aware loss, and pruning (Liu et al., 2025). Multimodal self-supervision addressed limited labels (Cao et al., 2025). Vision Transformer led a 2026 binary comparison of AlexNet, MobileNet, and U-Net (Menco-Tovar et al., 2026). Noise-tolerant experiments confirmed strong dependence on preprocessing and classifier choice (Nancy and Kiran, 2026).

Existing approaches demonstrate promising performance for either classification or segmentation; however, few simultaneously optimize both tasks within a unified architecture while integrating Transformer-based global context modeling, disease-guided feature interaction, and boundary-aware refinement. These limitations motivated the development of the proposed CuFormer U-Net.

## 3 Materials and methods

### 3.1 Overall workflow

The study uses two distinct tasks and data sources. The 3,754-image Kaggle dataset supports mutually exclusive three-class image classification (healthy, powdery mildew, and downy mildew). The separate greenhouse collection supports binary segmentation of visible symptomatic tissue, not pathogen-confirmed multi-class lesion segmentation. A 500-image greenhouse acquisition pool was screened into a 400-image development cohort (200 healthy and 200 affected leaves) and a separate 100-image condition-shift stress-test cohort. The development cohort was partitioned at the source-image level into 70% training, 15% validation, and 15% testing subsets; all derived crops and augmentations inherited the source-image split. Plant identity and acquisition-session identifiers were not retained in the available record, so stricter plant-level or session-level separation cannot be verified retrospectively. The overall workflow of the proposed CuFormer U-Net framework is shown in **Figure 1**.

**Figure 1:**
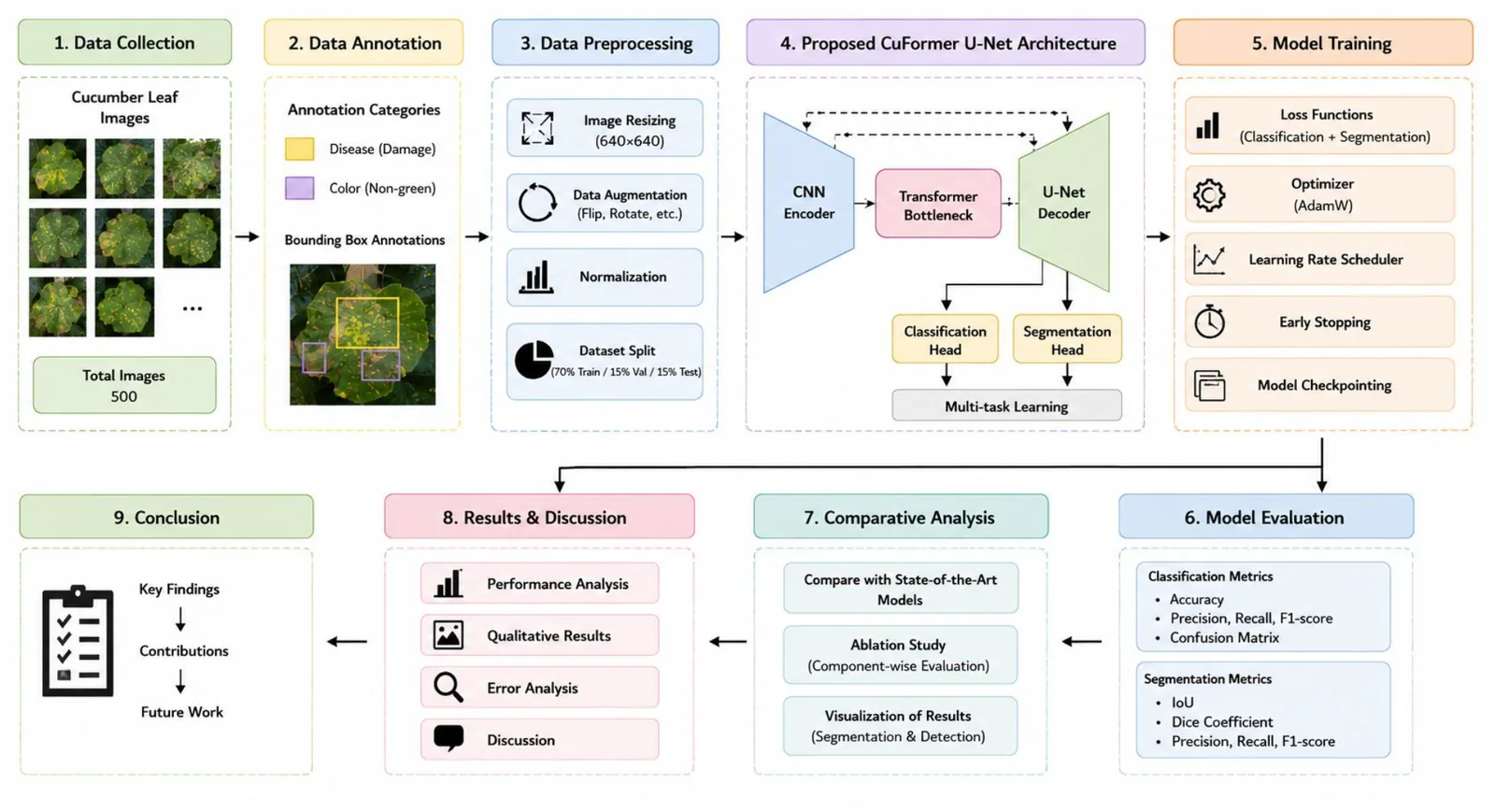
Overall workflow of the proposed CuFormer U-Net framework for cucumber leaf disease detection and segmentation.

Images were placed on a 640 × 640 annotation canvas and converted to 512 × 512 model inputs. Geometric and photometric augmentation was confined to the training split. AdamW optimization, cosine scheduling, early stopping, checkpoint selection, and metric implementations were applied consistently to the proposed model and in-study baselines. Exact augmentation probabilities and ranges were not preserved in the available experiment record and are therefore not reconstructed in this revision.

### 3.2 Dataset acquisition

RGB images were collected in greenhouse rows under natural and supplemental illumination. Acquisition included frontal and oblique viewpoints, overlapping leaves, irrigation equipment, support strings, varying distances, shadows, specular reflections, and heterogeneous symptom density. Images with severe motion blur, near-duplicate composition, or insufficient visible leaf area were excluded. The 400-image development cohort contained 200 healthy and 200 affected leaves. The affected subset contained 2,705 rectangle annotations: 1,741 color-abnormality instances and 964 tissue-damage instances. Healthy images contained no lesion annotation files.

The Kaggle dataset was retained as a separate public classification benchmark with three mutually exclusive categories: healthy, powdery mildew, and downy mildew. Each image receives one class label; multi-label disease co-occurrence is not represented in this benchmark. The public images were never mixed with the greenhouse segmentation test set. The greenhouse task instead predicts a binary mask of visible symptomatic tissue and does not assign pathogen-specific pixel labels.

### 3.3 Data collection and annotation

The public Cucumber Leaf Disease Dataset hosted on Kaggle contains 3,754 RGB images in three image-level categories: healthy, powdery mildew, and downy mildew. The primary classification task is therefore three-class, single-label recognition. The nominal source resolution is 1,366 × 768 pixels; all images were resized to 512 × 512 for model input.

The Kaggle images were used only for image-level classification. The greenhouse development cohort was used for binary visible-symptom segmentation after rectangle-seeded masks were manually refined. These tasks were evaluated separately because the public class labels and greenhouse spatial labels have different taxonomies and supervision levels. Derived samples were never allowed to cross a source-image split boundary.

This design prevents the public classification results from being attributed to the smaller greenhouse cohort. It also prevents the five-category exploratory symptom-crop analysis in **Figure 2** from being confused with the primary three-class Kaggle benchmark; **Figure 2** is retained only as a secondary diagnostic error analysis and is not used to calculate the headline classification metrics.

**Figure 2:**
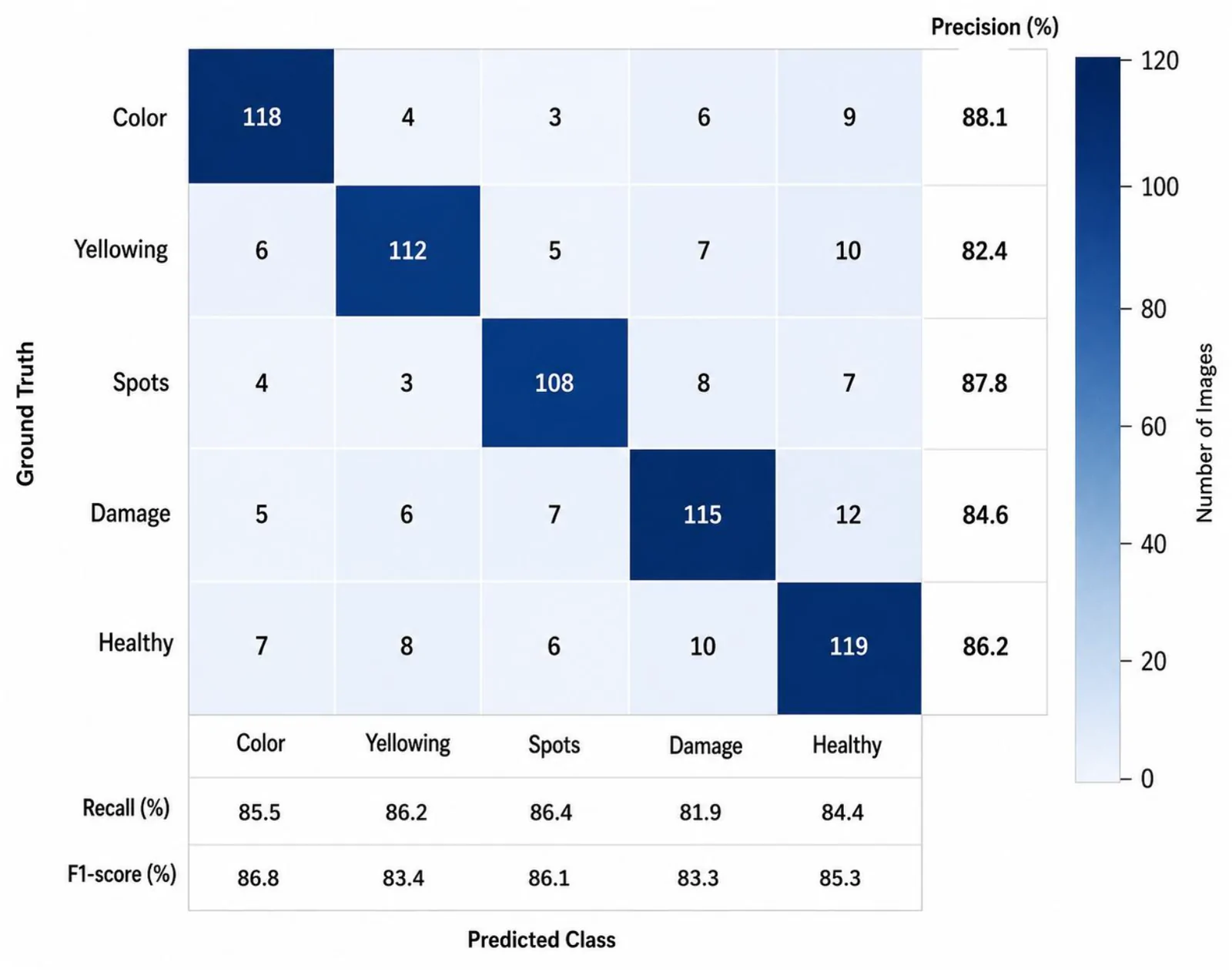
Exploratory five-category symptom-crop confusion matrix; this secondary analysis is separate from the primary three-class Kaggle benchmark.

**Table 1** reconciles the composition, label type, and experimental role of each data source. The 100-image greenhouse cohort is described as a condition-shift stress test rather than formal external validation because the available record does not preserve its class distribution or quantitative prediction outputs.

**Table 1:** Dataset composition, label definitions, partitioning, and experimental roles.

Data source	Composition	Labels/task	Split and reporting status
Kaggle classification benchmark	3,754 images; healthy, powdery mildew, downy mildew	One mutually exclusive image-level class; three-class classification	Source-image split; 512 × 512 model input; primary classification benchmark
Greenhouse development cohort	400 images: 200 healthy and 200 affected	2,705 rectangles (1,741 color abnormality; 964 tissue damage); rectangle-seeded masks manually refined; binary visible-symptom segmentation	70/15/15 source-image split; derived crops and augmentations inherit source split; plant/session grouping unavailable
Greenhouse condition-shift stress test	100 images with additional viewpoint, illumination, and occlusion variation	Intended stress test; exact class distribution not retained in the available manuscript record	No quantitative outputs available; not treated as formal external validation

### 3.4 Annotation protocol and quality control

Rectangle annotations were created for two visible-phenotype categories: color abnormality and tissue damage. These categories describe appearance and are not pathogen-confirmed disease classes. Rectangle-seeded candidate masks were manually refined to obtain the binary foreground masks used for segmentation. However, the available annotation record does not identify the number or expertise of annotators, retain independent annotator masks, document adjudication events, or provide an inter-annotator agreement statistic. These details cannot be reconstructed responsibly and should be completed from the original annotation log before resubmission.

### 3.5 Partitioning and leakage prevention

The split unit was the original source image. All augmentations, symptom-centered crops, and mask derivatives inherited that source-image identifier. The public and greenhouse datasets remained disjoint. Because plant, leaf-instance, greenhouse-location, and acquisition-session identifiers were not preserved in the archived manuscript record, the revision does not claim verified separation at those higher grouping levels. This residual leakage risk is now stated explicitly.

### 3.6 External greenhouse condition-shift cohort

The additional 100 greenhouse images were selected to increase viewpoint, illumination, occlusion, and background variation. They were not used for training or checkpoint selection. The available files do not contain the cohort’s healthy/affected distribution, per-condition strata, predictions, or aggregate metrics. Consequently, this cohort is treated as a reserved qualitative stress test rather than an external validation set, and no numerical generalization claim is based on it.

### 3.7 Proposed CuFormer U-net

CuFormer U-Net was designed as a unified multi-task framework that combines efficient convolutional feature extraction with global contextual modeling for simultaneous disease recognition and lesion segmentation. The architecture emphasizes multi-scale representation learning and task interaction so that classification information can guide spatial lesion localization while preserving fine symptom boundaries.

**Figure 3** presents a simplified view of the proposed architecture. A convolutional stem and hierarchical encoder retain local texture; a multi-scale context module feeds a compact 64-token Transformer bottleneck; classification probabilities form a disease embedding; and that embedding both modulates bottleneck features and conditions attention-guided skip transfer. A residual decoder, deep supervision, and boundary refinement produce a binary visible-symptom mask, while a separate head produces the three-class image label. **Figures 4**, **5** retain the component-level details. The directed classification-to-segmentation guidance is the principal distinction from architectures that merely attach independent heads to a shared encoder.

**Figure 3:**
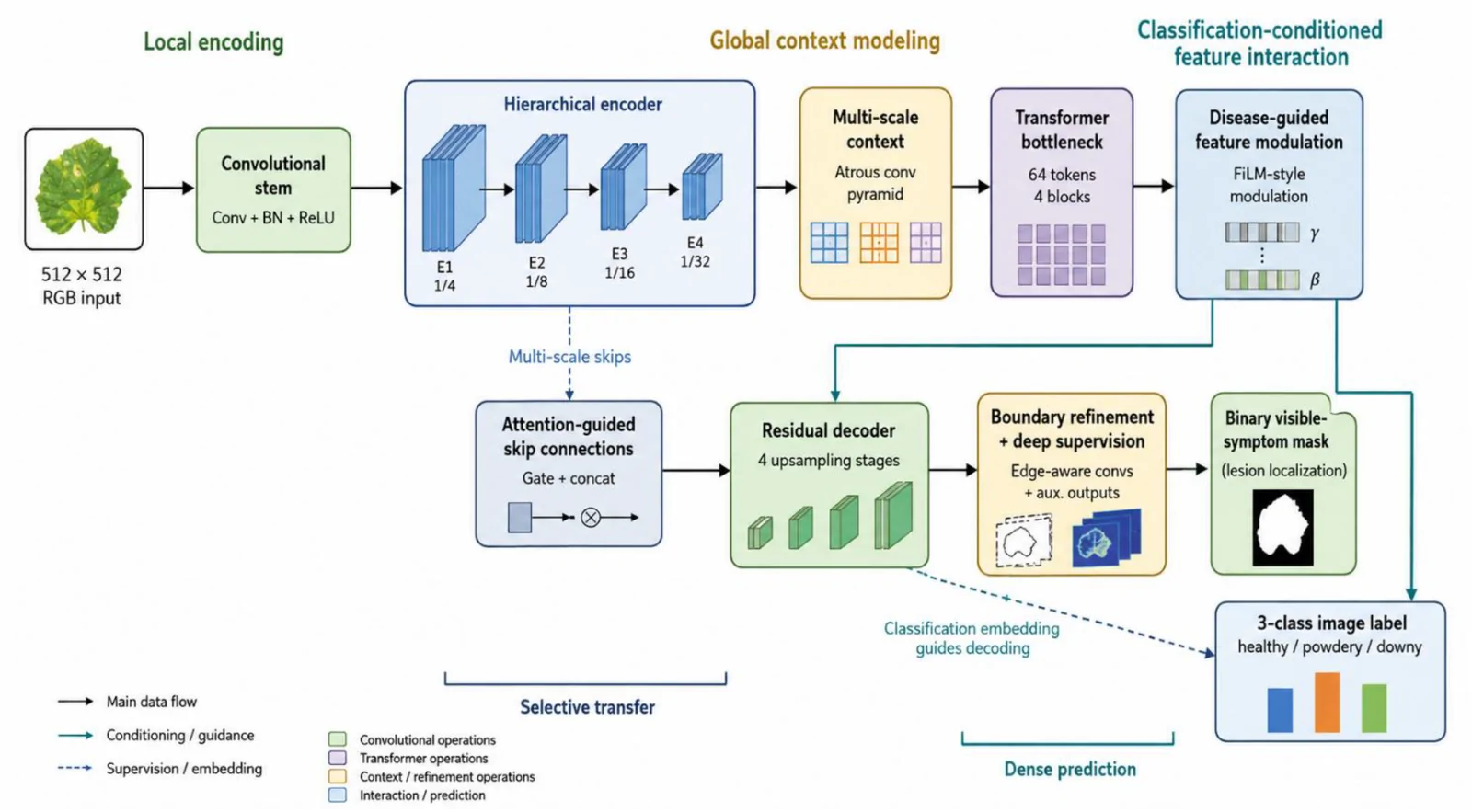
Overall architecture of the proposed CuFormer U-Net.

**Figure 4:**
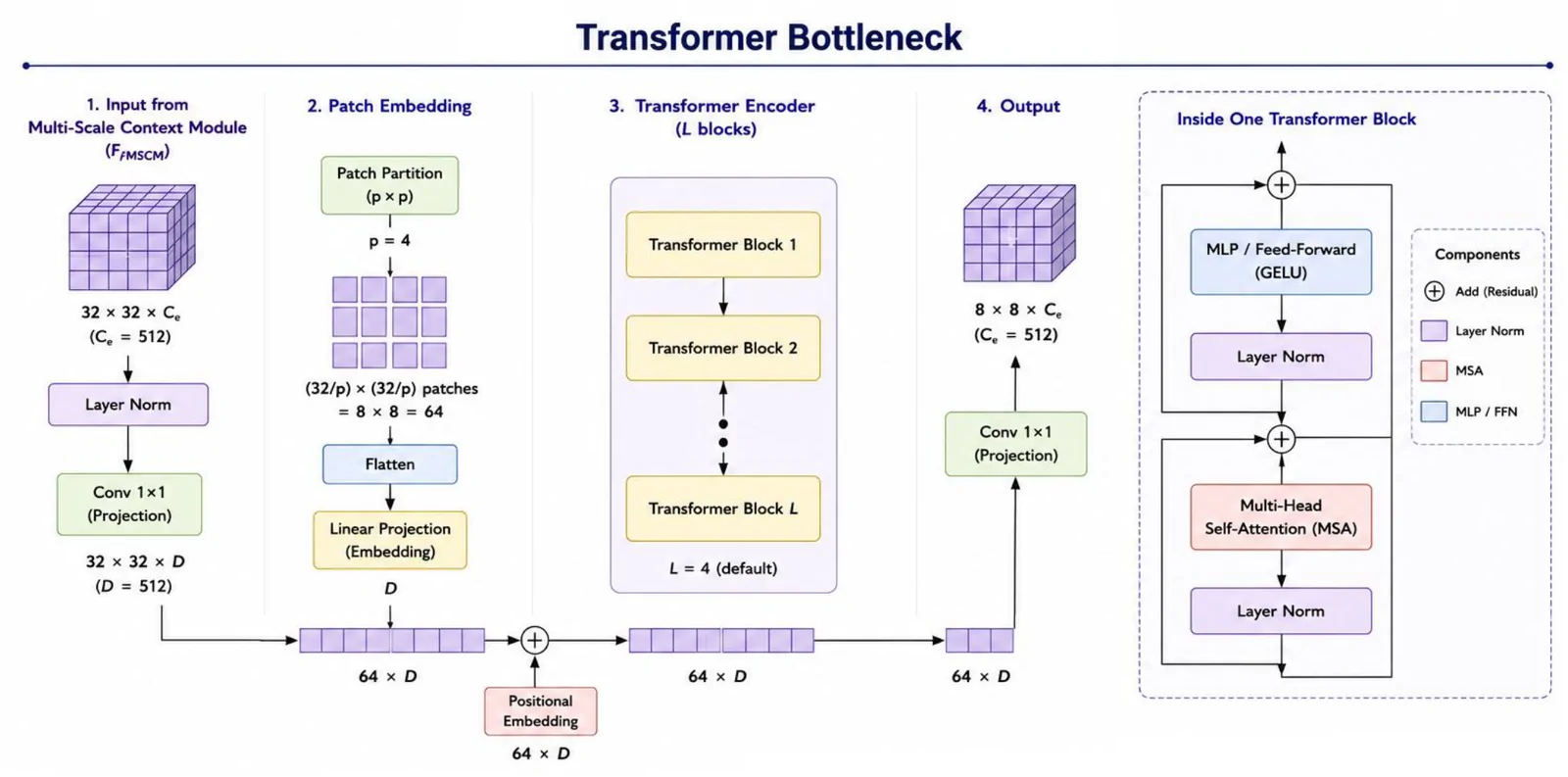
Transformer bottleneck.

**Figure 5:**
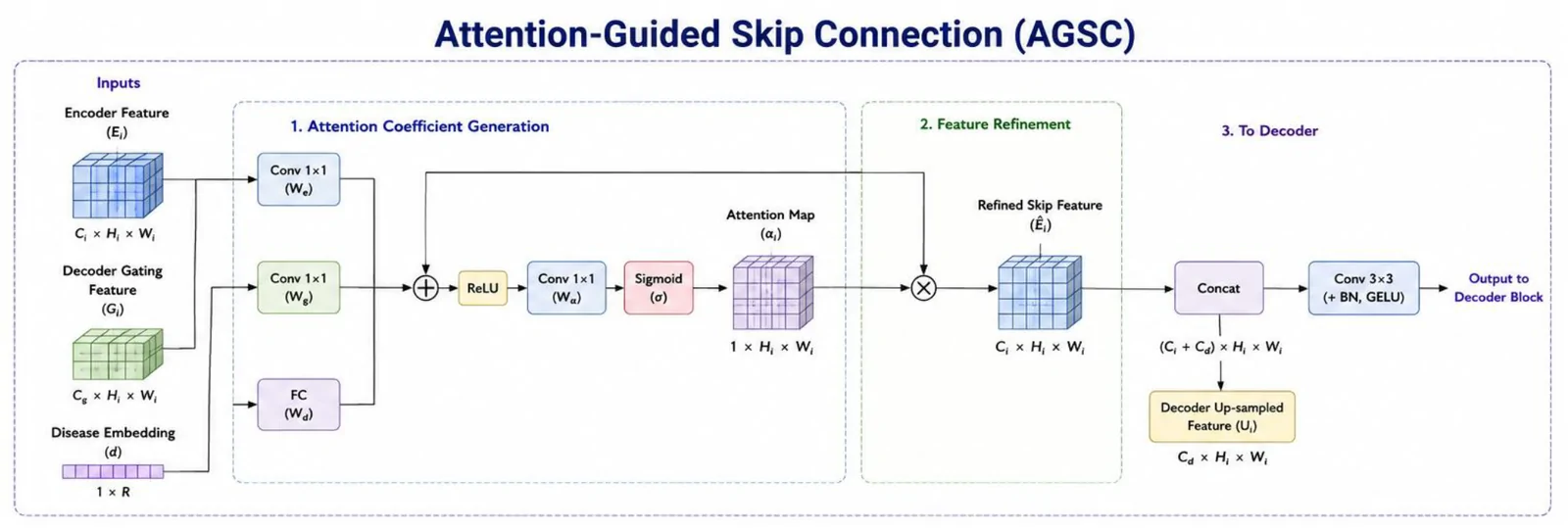
Attention-guided skip connection.

#### 3.7.1 Convolutional stem

For input $X\in {\mathbb{R}}^{512\times 512\times 3}$, the stem applies two 3 × 3 convolutions with batch normalization and Gaussian error linear unit activation:


$$F_{0}=\text{GELU }(\text{BN}(W_{2}*\text{GELU}(\text{BN}(W_{1}*X)))).$$

The resulting 512×512×64 tensor preserves edge and color transitions before hierarchical downsampling.

#### 3.7.2 Hierarchical encoder

The encoder follows ConvNeXt-Tiny stage principles because modernized depthwise convolution provides strong local inductive bias and competitive scaling efficiency (Liu et al., 2022a). Four stages generate $E_{1}\in {\mathbb{R}}^{256\times 256\times 64}$, $E_{2}\in {\mathbb{R}}^{128\times 128\times 128}$, $E_{3}\in {\mathbb{R}}^{64\times 64\times 256}$, and $E_{4}\in {\mathbb{R}}^{32\times 32\times 512}$. Each block uses a 7 × 7 depthwise convolution, layer normalization, pointwise expansion, GELU, pointwise projection, and a residual path. Stochastic depth regularizes deeper stages.

#### 3.7.3 Multi-scale context module

The multi-scale context module applies parallel 3 × 3 dilated convolutions with dilation rates r∈{1,3,6,9} and a global pooling branch. Their outputs are concatenated and projected:


$$F_{MSCM}=W_{p}*[\phi_{1}(E_{4});\phi_{3}(E_{4});\phi_{6}(E_{4});\phi_{9}(E_{4});U(W_{g}*\text{GAP}(E_{4}))],$$

where [·] denotes channel concatenation, U denotes upsampling, and $W_{p}$ is a 1 × 1 projection. This module preserves local spot texture while representing larger yellowing or necrotic regions.

#### 3.7.4 Transformer bottleneck

At the deepest stage of CuFormer U-Net, the Transformer bottleneck is introduced to capture long-range dependencies that may be difficult to represent using local convolutional operations alone. The bottleneck converts the compact spatial feature map into a sequence of tokens while preserving information about their spatial organization. Multi-head self-attention then enables interactions among distant lesion regions and contextual leaf features. This design complements the encoder’s local representations with global contextual information before features are passed to the decoder. **Figure 4** demonstrates the bottleneck transformation. The 32×32×512 context tensor is normalized and partitioned into non-overlapping 4 × 4 patches, producing 64 tokens of dimension 512. Learned positional embeddings retain patch order. Four pre-normalized Transformer blocks update the token matrix Z:


$$Z{"}_{l}=Z_{l-1}+\text{MSA}(\text{LN}(Z_{l-1})),$$

$$Z_{l}=Z{"}_{l}+\text{MLP}(\text{LN}(Z{"}_{l})),$$

l=1,…,4.

For each attention head, scaled dot-product attention is.


$$\text{Attn}(Q,K,V)=\text{softmax}(\frac{QK^{⊤}}{\sqrt{d_{k}}})V.$$

The 8 × 8 token grid is projected, reshaped, and upsampled to a 32×32×512 spatial feature map. Restricting attention to the deepest scale reduces token count from 16,384 at 512 × 512 with 4 × 4 patches to 64, making global modeling tractable.

#### 3.7.5 Disease-guided feature modulation

The classification head first predicts $\hat{p}=\text{softmax}(W_{c}\text{GAP}(F_{T}))$, where $F_{T}$ is the reconstructed Transformer feature. A learned projection converts $\hat{p}$ into disease embedding $d=W_{d}\hat{p}$. The embedding is broadcast across spatial positions, concatenated with $F_{T}$, and transformed by a 1 × 1 convolution:


$$F_{DG}=W_{m}*[F_{T};\text{Broadcast}(d)].$$

This mechanism encourages the dense decoder to emphasize spatial patterns compatible with the current diagnostic evidence while remaining differentiable under joint training.

#### 3.7.6 Attention-guided skip connections

Attention-guided skip connections were incorporated to selectively transfer diagnostically relevant encoder features to the decoder while suppressing background responses. Unlike conventional direct skip connections, the proposed mechanism evaluates encoder information according to both the current decoder context and the predicted disease representation. This conditioning allows the network to emphasize spatial regions associated with visible symptoms, including discoloration, necrotic tissue, and irregular lesion boundaries. The resulting attention coefficients adaptively regulate the contribution of encoder features before feature fusion. Consequently, the decoder receives more task-relevant spatial information for accurate lesion reconstruction and boundary delineation.

**Figure 5** depicts an attention-guided skip connection. Encoder feature $E_{i}$, decoder gating feature $G_{i}$, and disease embedding d jointly generate a spatial coefficient:


$$\alpha_{i}=\sigma (W_{\alpha }*\text{ReLU}(W_{e}*E_{i}+W_{g}*G_{i}+W_{d}d)),$$

$${\hat{E}}_{i}=\alpha_{i}⊙E_{i}.$$

The refined feature ${\hat{E}}_{i}$ is concatenated with the upsampled decoder feature and processed by a 3 × 3 convolution, batch normalization, and GELU. Unlike direct U-Net concatenation, this operation suppresses background foliage, structural veins, and greenhouse equipment when they are inconsistent with the decoder state and disease embedding.

#### 3.7.7 Residual decoder

The decoder contains four stages $D_{4},D_{3},D_{2},D_{1}$ at resolutions 32, 64, 128, and 256 pixels. Each stage upsamples the preceding representation, concatenates its gated skip feature, and applies a two-convolution residual block. A final upsampling operation restores 512 × 512 output resolution. Residual decoding maintains gradient flow and limits the loss of weak lesion evidence during reconstruction.

#### 3.7.8 Boundary-aware refinement

The boundary branch fuses shallow features $E_{1}$, resized $E_{2}$, and decoder feature $D_{1}$. The binary boundary target is generated from the ground-truth mask M by morphological dilation and erosion:


B=Dilate(M)−Erode(M).

A 3 × 3 refinement block predicts $\hat{B}$, which is added to the final decoder logits before the segmentation activation. This auxiliary branch concentrates supervision on narrow transition regions rather than allowing easy interior pixels to dominate learning.

#### 3.7.9 Classification head

Global average pooling converts $F_{T}$ into a 512-dimensional vector. A fully connected layer, GELU, dropout of 0.3, and a final C-unit layer output disease probabilities. C=3 for the Kaggle healthy/powdery-mildew/downy-mildew benchmark and C=5 for the greenhouse symptom-crop diagnostic analysis. Adjusting only the last layer preserves the shared representation and experimental comparability.

#### 3.7.10 Segmentation head

The final segmentation head applies a 1 × 1 convolution to the refined decoder output and uses a sigmoid for binary lesion segmentation. Intermediate heads at three decoder scales are upsampled to full resolution for deep supervision. The predicted lesion area supports severity estimation through $S=100|\hat{M}|/|L|$, where L denotes the segmented leaf region.

### 3.8 Loss functions

Classification uses weighted cross-entropy with label smoothing ϵ=0.05. Segmentation combines binary cross-entropy and soft Dice loss. Boundary prediction uses binary cross-entropy, while intermediate masks use the same segmentation objective at reduced weight. The Dice loss is.


$$L_{Dice}=1-\frac{2\sum_{j}p_{j}g_{j}+\epsilon }{\sum_{j}p_{j}+\sum_{j}g_{j}+\epsilon }.$$

The total objective is.


$$L=\lambda_{c}L_{cls}+\lambda_{s}(0.5L_{BCE}+0.5L_{Dice})+\lambda_{b}L_{bound}+\lambda_{d}\sum_{k=1}^{3}2^{-k}L_{seg}^{\left(k\right)},$$

with $\left(\lambda_{c},\lambda_{s},\lambda_{b},\lambda_{d})=(1.0,1.0,0.3,0.4\right)$. Gradient norms were monitored to ensure that neither classification nor segmentation dominated shared-encoder updates.

### 3.9 Experimental setup

Models were implemented in PyTorch and trained for a maximum of 200 epochs using AdamW, an initial learning rate of 1 × 10−4, weight decay of 1 × 10−4, five warm-up epochs, cosine annealing, batch size eight, and mixed-precision training. Early stopping used a 20-epoch patience based on the mean of validation macro F1 and Dice. The encoder used ImageNet-pretrained weights and newly introduced layers used truncated-normal initialization. Experiments used an NVIDIA RTX 3090 GPU with 24 GB memory and 64 GB system RAM. The exact original random seed, truncated-normal standard deviation, and probabilities and numerical ranges for each augmentation were not retained in the available experiment record. They are not invented in this revision. Exact reproduction and independent-seed reruns therefore require the archived training configuration or a confirmatory rerun.

Classification accuracy and macro precision, recall, and F1 were computed across the three mutually exclusive Kaggle classes. For segmentation, precision, recall, and IoU were computed for the visible-symptom foreground, while macro IoU denotes an unweighted average across foreground and background. Dice was calculated per image and averaged across images. Pixel-level F1 equals Dice only when both are calculated from the same pooled counts and aggregation scheme; because the manuscript previously mixed image-wise Dice and pooled F1, the redundant segmentation F1 interpretation has been removed from the principal claims. TP, FP, FN, and TN refer to thresholded pixel predictions at 0.5.


$$\text{Precision}=\frac{TP}{TP+FP},$$

$$\text{Recall}=\frac{TP}{TP+FN},$$

$$F_{1}=\frac{2TP}{2TP+FP+FN},$$

$$IoU=\frac{TP}{TP+FP+FN}.$$

All in-study baselines used the same source-image split, 512 × 512 input resolution, preprocessing pipeline, training-only augmentation policy, AdamW optimizer family, 200-epoch ceiling, early-stopping rule, checkpoint criterion, and metric implementation. Classification backbones used comparable ImageNet initialization. Segmentation baselines were trained with the same binary masks and standard decoders. The available record does not contain a complete model-specific hyperparameter-search log; comparisons are therefore described as controlled training comparisons rather than exhaustive best-possible tuning for every baseline.

### 3.10 Statistical analysis and reproducibility scope

The reported tables contain fixed-split point estimates from the archived experiment record. Independent runs with different seeds, run-level standard deviations, per-image prediction vectors, confidence intervals, and paired significance tests were not available. Consequently, no p-values or claims of statistical significance are reported. Differences are expressed descriptively in percentage points. A confirmatory protocol should rerun every proposed and baseline model with the same five prespecified seeds (17, 42, 73, 101, and 2026), report mean ± standard deviation and 95% confidence intervals, and apply paired tests to identical test images with multiplicity correction. These seed values define the proposed confirmatory protocol; they are not presented as seeds used for the current point estimates.

### 3.11 Computational profiling scope

The manuscript record identifies the training GPU but does not retain a parameter-count report, FLOP trace, latency benchmark, peak GPU memory measurement, or CPU specification. The previous complexity scatterplot could therefore not be verified and has been removed. Before resubmission, profiling should be repeated at batch size one and 512 × 512 input, with parameter count, multiply-accumulate convention, warm-up iterations, timed iterations, software versions, GPU/CPU models, and peak memory reported for CuFormer U-Net and the principal baselines.

## 4 Experimental results

### 4.1 Training convergence

The following results are fixed-split point estimates from the available experiment record. They support descriptive comparison but do not establish run-to-run variability or statistical significance. Internal classification and segmentation results are reported separately from the reserved 100-image greenhouse condition-shift cohort, for which quantitative outputs were unavailable.

**Figure 6** shows the recorded optimization trajectory for one run across 190 effective epochs. Training loss decreased from approximately 1.35 to below 0.10 and validation loss to approximately 0.14. Training accuracy approached 96%, validation accuracy approximately 94%, training Dice approximately 0.90, and validation Dice approximately 0.87. Because this is a single trajectory, it is evidence of convergence for that run only and does not quantify variability across random initialization or data-order effects.

**Figure 6:**
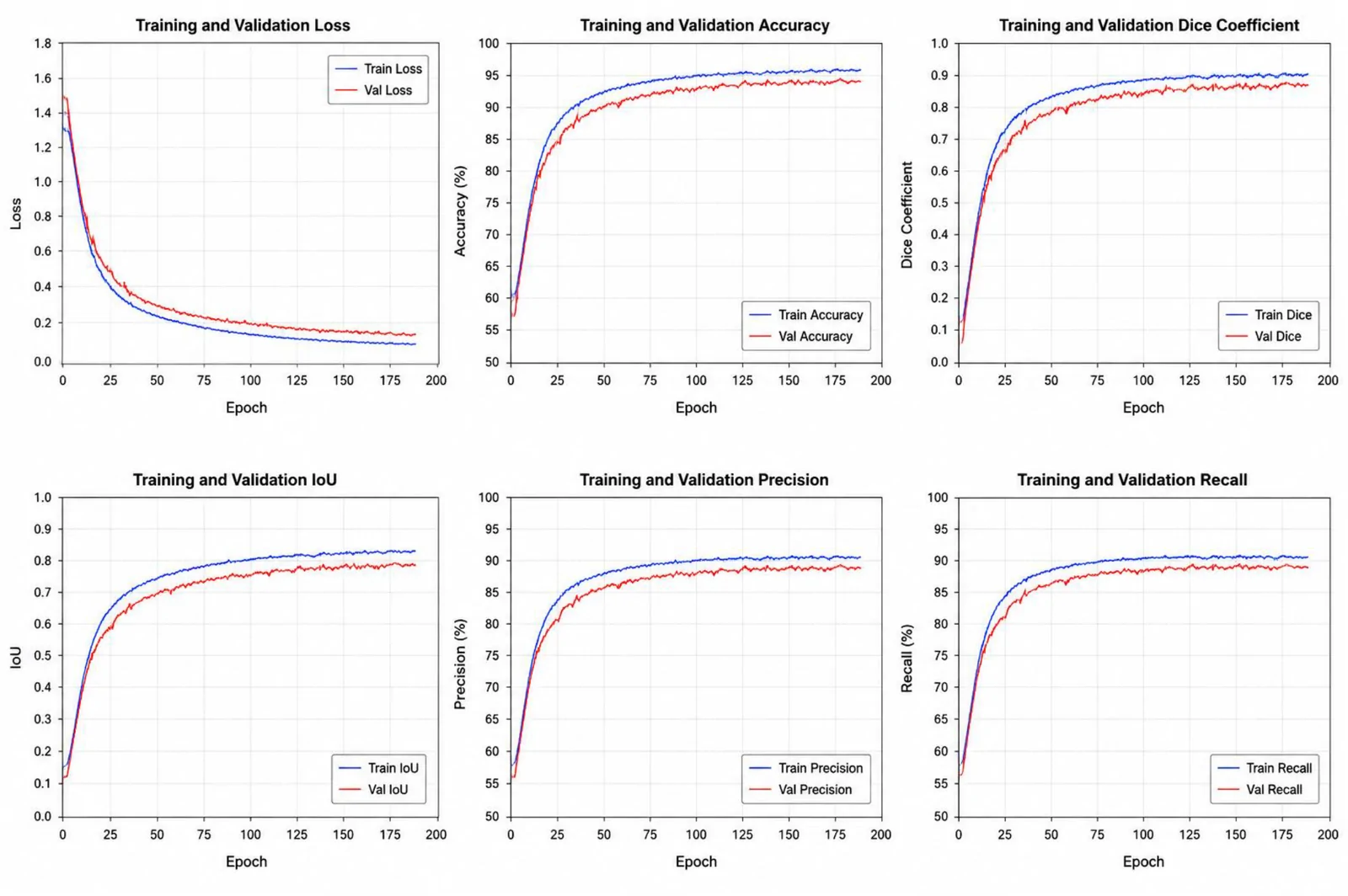
Training and validation performance of CuFormer U-Net across training epochs.

The rapid gains during the first 40 epochs reflect transfer from the pretrained encoder, while slower improvement after epoch 80 corresponds to boundary and task-interaction refinement. Validation curves remained noisier than training curves because a small number of difficult greenhouse images contained extensive occlusion, weak discoloration, or ambiguous damage.

### 4.2 External greenhouse stress-test reporting

The 100-image condition-shift cohort remained excluded from training and checkpoint selection. However, its class distribution and prediction outputs were not present in the supplied experiment record, so accuracy, Dice, IoU, precision, recall, and stratified illumination/occlusion results cannot be reported. The revision therefore removes claims that this cohort quantitatively demonstrates external generalization. A future external-validation table should report overall and condition-stratified metrics with confidence intervals after the cohort labels and predictions are recovered.

### 4.3 Classification performance

Image-level classification performance was evaluated to determine how effectively the proposed model distinguishes cucumber leaf conditions from visual symptom patterns. The comparison included representative convolutional, Transformer-based, and hybrid architectures under the same controlled evaluation protocol. Accuracy, precision, recall, and F1-score were selected as complementary metrics to capture overall predictive correctness and class-discrimination reliability. This benchmarking provides a direct assessment of whether the proposed integration of local, global, and lesion-aware representations improves classification performance over established architectures.

**Table 2** reports the canonical three-class classification point estimates: CuFormer U-Net achieved 96.7% accuracy, 96.3% macro precision, 95.8% macro recall, and 96.0% macro F1. CoAtNet-0 was the strongest in-study comparator at 94.4% accuracy and 93.8% macro F1, corresponding to descriptive differences of 2.3 and 2.2 percentage points. These values are used consistently in the abstract, **Figure 7**, discussion, and conclusion. Without independent reruns or paired per-image predictions, the differences are not characterized as statistically significant.

**Table 2:** Quantitative comparison of image-level cucumber leaf disease classification performance.

Method	Backbone	Accuracy (%)	Precision (%)	Recall (%)	F1-score (%)
ResNet18	CNN	86.1	85.7	84.9	85.3
ResNet50	CNN	88.8	89.3	88.2	88.8
ResNet101	CNN	91.2	90.7	90.1	90.4
DenseNet121	CNN	90.1	89.0	88.7	88.9
DenseNet169	CNN	91.4	90.8	90.2	90.5
VGG16	CNN	87.9	86.7	86.0	86.5
EfficientNet-B0	CNN	90.3	89.0	88.6	88.8
EfficientNet-B4	CNN	92.6	92.3	91.6	91.9
MobileNetV3-Large	Lightweight CNN	89.2	88.5	87.9	88.2
ConvNeXt-Tiny	CNN	93.1	92.7	92.0	92.3
Vision Transformer (ViT-B/16)	Transformer	93.5	93.2	92.8	93.0
Swin Transformer	Hierarchical Transformer	94.1	93.8	93.4	93.6
CNN-RNN-Attention	Hybrid	93.8	93.2	92.5	92.8
CoAtNet-0	CNN + Transformer	94.4	94.0	93.7	93.8
**CuFormer U-Net (proposed)**	**CNN + Transformer + multi-task**	**96.7**	**96.3**	**95.8**	**96.0**

Bold values indicate the best-performing result in each column.

**Figure 7:**
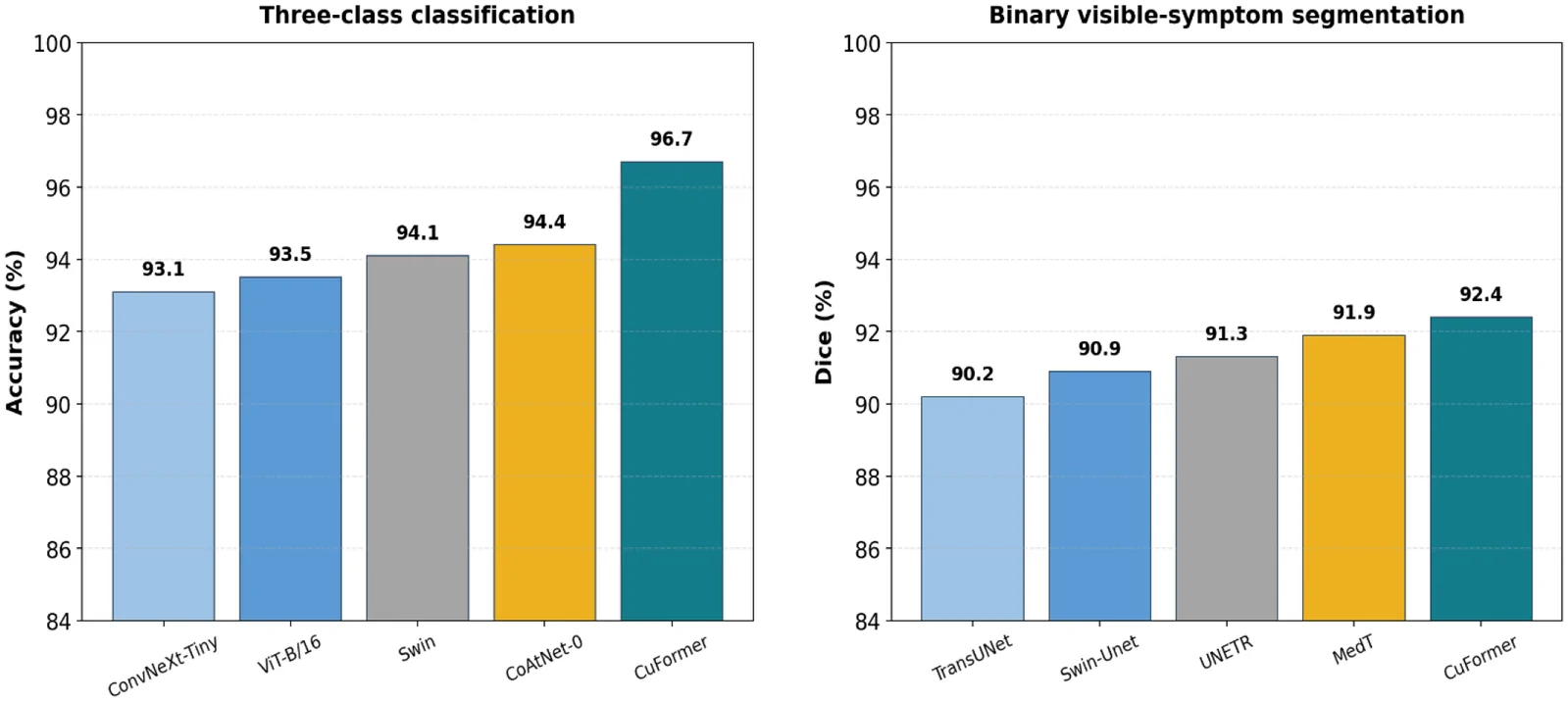
Canonical fixed-split classification accuracy and segmentation dice values.

**Figure 2** is a secondary five-category symptom-crop error analysis (Color, Yellowing, Spots, Damage, and Healthy), not the primary three-class Kaggle classification benchmark. The exploratory matrix contains 700 symptom-centered crops and is reported only to show visual confusions among phenotype-oriented crop labels. Its values are not combined with, or used to support, the 96.7% three-class image-level accuracy reported in **Table 2**.

### 4.4 Segmentation performance

Lesion segmentation performance was evaluated to determine the ability of each model to accurately localize visible symptomatic regions at the pixel level. The qualitative comparison considered leaves exhibiting substantial variation in lesion size, distribution, morphology, and boundary complexity. Particular attention was given to false-positive suppression, preservation of small affected regions, and accurate delineation of irregular symptom boundaries. These visual comparisons complement the quantitative metrics by demonstrating how effectively the competing architectures reconstruct disease-affected areas under heterogeneous symptom presentations.

**Figure 8** illustrates qualitative results for five affected leaves with distinct symptom densities and shapes. U-Net produced numerous false-positive fragments and irregular interiors. U-Net++ improved continuity but retained scattered errors. Attention U-Net suppressed some irrelevant pixels, while DeepLabV3+ represented larger affected regions more consistently. TransUNet captured distributed lesions but occasionally expanded disease beyond true boundaries. CuFormer U-Net most closely followed the ground-truth extent across sparse spots, dense lesions, edge damage, and large necrotic regions.

**Figure 8:**
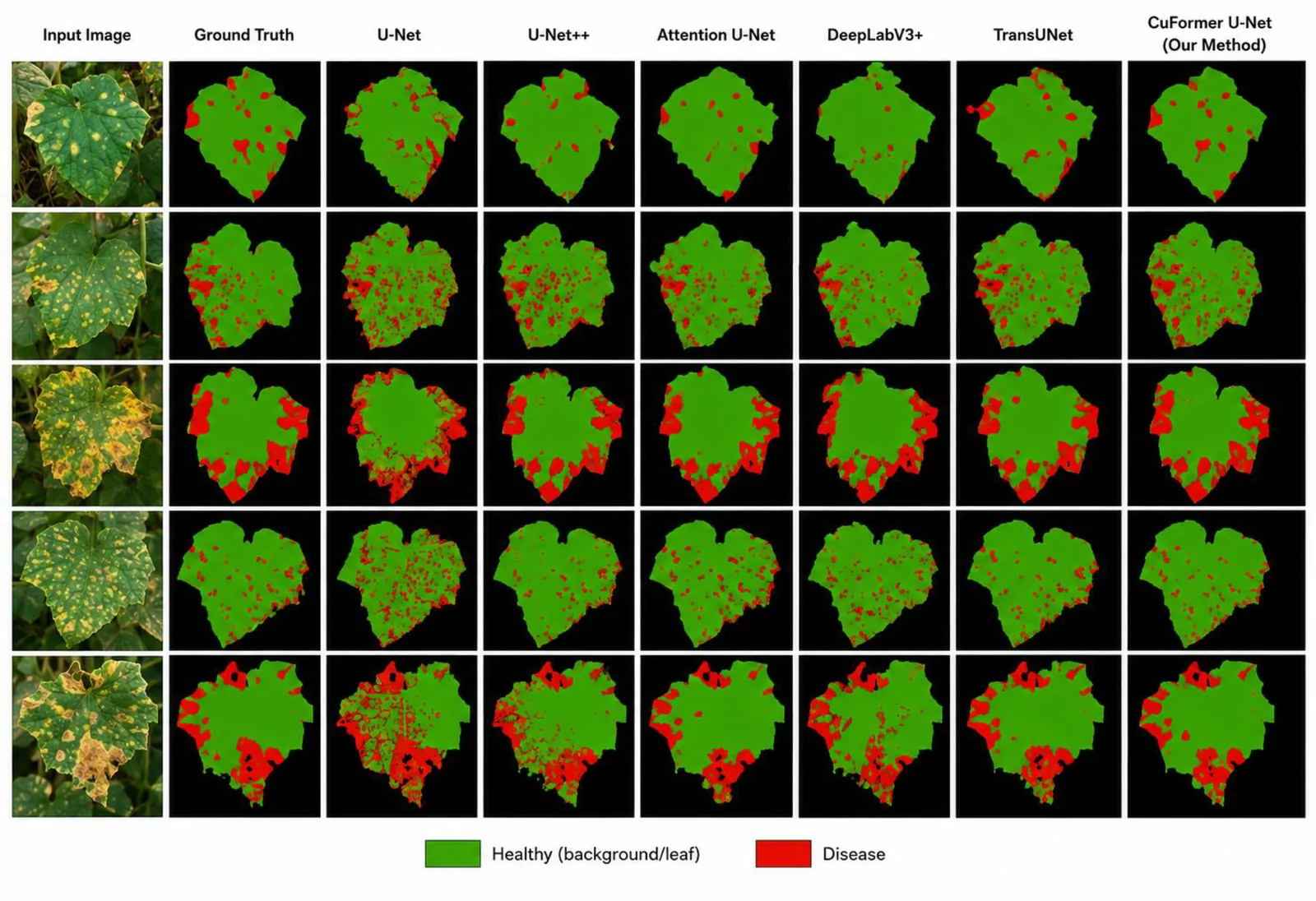
Qualitative comparison of disease segmentation results obtained by CuFormer U-Net and baseline models.

**Table 3** reports the canonical segmentation point estimates: CuFormer U-Net achieved 92.4% mean image-wise Dice, 83.1% macro IoU, 93.2% foreground precision, and 91.7% foreground recall. MedT was the strongest listed comparator at 91.9% Dice. The 0.5-point Dice difference is descriptive only because run-level variability and paired predictions were unavailable. The 88.6% foreground-restricted IoU reported in the previous version used a different aggregation from macro IoU and has been removed from headline cross-model claims to avoid conflating definitions.

**Table 3:** Quantitative comparison of cucumber leaf disease segmentation performance.

Method	Architecture	Dice (%)	IoU (%)	Precision (%)	Recall (%)	F1-score (%)
FCN-8s	Fully convolutional network	74.3	63.2	76.5	72.2	73.3
SegNet	Encoder-decoder CNN	77.2	66.8	79.4	75.6	76.9
PSPNet	Pyramid scene parsing	81.6	70.4	83.8	80.2	81.5
U-Net	Encoder-decoder	83.5	72.8	84.3	82.1	83.2
U-Net++	Nested skip connections	86.6	75.9	86.2	85.1	85.6
Attention U-Net	Attention gates	88.7	78.4	88.4	87.3	87.8
DeepLabV3+	Atrous spatial pyramid pooling	89.8	79.9	89.9	88.2	89.0
HRNetV2	High-resolution network	90.1	80.3	90.0	88.7	89.3
TransUNet	CNN + Transformer	90.2	80.7	90.6	89.5	90.0
Swin-Unet	Hierarchical Transformer	90.9	81.4	91.0	89.9	90.4
UNETR	Transformer encoder	91.3	82.0	91.4	90.2	90.8
SegFormer-B2	MiT Transformer	91.7	82.5	91.8	90.8	91.2
Mask R-CNN	Instance segmentation	87.2	76.5	87.4	86.1	86.7
MedT	Medical Vision Transformer	91.9	82.8	92.0	90.9	91.4
**CuFormer U-Net (proposed)**	**CNN + Transformer + attention + disease guidance**	**92.4**	**83.1**	**93.2**	**91.7**	**92.4**

Bold values indicate the best-performing result in each column.

### 4.5 Comparison with published cucumber disease studies

Comparison with previously published studies provides broader context for evaluating the classification capability of CuFormer U-Net beyond the controlled experiments conducted in this work. However, cross-study performance should be interpreted carefully because the reported experiments differ substantially in dataset composition, class definitions, augmentation strategies, train-test partitions, preprocessing procedures, and evaluation conditions. Some studies retained the original three-class Kaggle formulation, whereas others merged additional datasets, reformulated the task as binary classification, introduced additional disease categories, or evaluated robustness after synthetic noise injection. Such methodological differences can substantially affect task complexity and reported accuracy, preventing a strictly equivalent numerical ranking of the models. Accordingly, the comparison emphasizes both reported predictive performance and the experimental setting in which each result was obtained. An additional distinction of the proposed approach is its multi-task formulation, which jointly learns image-level disease classification and lesion segmentation rather than optimizing classification alone. This comparison therefore positions CuFormer U-Net relative to existing cucumber disease recognition approaches while preserving appropriate consideration of differences among experimental protocols.

**Table 4** demonstrates substantial variation in reported cucumber disease classification performance across architectures and experimental protocols. Within the original three-class setting, Shahid and Zohaib reported accuracies of 92.15%, 98.76%, 97.43%, and 97.93% for the custom CNN, VGG16, InceptionV3, and ResNet50, respectively, while Gill et al. obtained 91.00% using transfer-learning ResNet50. The highest reported accuracy, 99.39%, was achieved by Vision Transformer in the binary experiment of Menco-Tovar et al.; however, this result was obtained after combining datasets and reducing the task to healthy-versus-diseased classification, limiting direct comparability. Their MobileNet similarly achieved 99.00%, whereas AlexNet and the U-Net classifier showed substantially weaker results. Under a modified five-class protocol, Bilal et al. reported 97.28% accuracy. Noise-oriented experiments by Nancy and Kiran varied markedly from 32% to 96%, demonstrating sensitivity to classifier selection and experimental conditions. CuFormer U-Net achieved 96.70% accuracy, 96.30% precision, 95.80% recall, and a 96.00% F1-score on the original three-class dataset. Although some classification-only studies reported higher accuracy, the proposed framework provides the additional capability of jointly learning disease classification and lesion segmentation, offering both diagnostic prediction and spatial characterization of affected tissue within a unified model.

**Table 4:** Performance comparison of CuFormer U-Net with published experiments using the Kaggle Cucumber Leaf Disease Dataset.

Study	Architecture or classifier	Dataset protocol	Classification setting	Reported quantitative result
Shahid and Zohaib (2025)	Custom CNN	Original three classes followed by augmentation; 70:30 training-testing split	Healthy, powdery mildew, downy mildew	Accuracy: 92.15%
Shahid and Zohaib (2025)	VGG16	Original three classes followed by augmentation; 70:30 split	Three-class classification	Accuracy: 98.76%
Shahid and Zohaib (2025)	InceptionV3	Original three classes followed by augmentation; 70:30 split	Three-class classification	Accuracy: 97.43%
Shahid and Zohaib (2025)	ResNet50	Original three classes followed by augmentation; 70:30 split	Three-class classification	Accuracy: 97.93%
Gill et al. (2025)	Transfer-learning ResNet50	Original 3,754-image dataset	Three-class classification	Accuracy: 91.00%
Menco-Tovar et al. (2026)	AlexNet	Dataset combined with a second Kaggle cucumber dataset; 70:15:15 split	Binary healthy-diseased classification	Accuracy: 63.00%; precision: 62.97%; recall: 64.37%; F1: 39.00%; AUC: 50.00%
Menco-Tovar et al. (2026)	Vision Transformer	Combined Kaggle datasets; 70:15:15 split	Binary healthy-diseased classification	Accuracy: 99.39%; precision: 99.76%; recall: 99.28%; F1: 99.52%; AUC: 99.43%
Menco-Tovar et al. (2026)	MobileNet	Combined Kaggle datasets; 70:15:15 split	Binary healthy-diseased classification	Accuracy: 99.00%; precision: 99.75%; recall: 98.26%; F1: 99.00%; AUC: 98.92%
Menco-Tovar et al. (2026)	U-Net classifier	Combined Kaggle datasets; 70:15:15 split	Binary healthy-diseased classification	Accuracy: 78.23%; precision: 76.90%; recall: 61.90%; F1: 68.59%; AUC: 84.98%
Bilal et al. (2025)	Fuzzy deep CNN with chaotic PSO	Original Kaggle source supplemented with two additional classes	Modified five-class classification	Test-1 accuracy: 97.28%; false-negative rate: 2.72%
Nancy and Kiran (2026)	LGBM with Gaussian filtering	Kaggle-derived five-class dataset with synthetic Gaussian noise; 70:30 split	Noise-tolerant multiclass classification	Accuracy: 32.00%; precision: 24.00%; recall: 30.00%; F1: 23.00%
Nancy and Kiran (2026)	Modified Random Forest with Gaussian filtering	Kaggle-derived five-class dataset with synthetic Gaussian noise; 70:30 split	Noise-tolerant multiclass classification	Accuracy: 96.00%; precision: 96.00%; recall: 95.00%; F1: 95.00%
Nancy and Kiran (2026)	QSVM with Gaussian filtering	Kaggle-derived five-class dataset with synthetic Gaussian noise; 70:30 split	Noise-tolerant multiclass classification	Accuracy: 35.00%; precision: 34.00%; recall: 37.00%; F1: 28.00%
Nancy and Kiran (2026)	Multi-SVM with Gaussian filtering	Kaggle-derived five-class dataset with synthetic Gaussian noise; 80:20 split	Noise-tolerant multiclass classification	Accuracy: 63.00%; precision: 85.00%; recall: 63.00%; F1: 69.00%
**Proposed study**	**CuFormer U-Net**	**Original three-class Kaggle dataset; classification and lesion segmentation trained jointly**	**Three-class multi-task classification**	**Accuracy: 96.70%; precision: 96.30%; recall: 95.80%; F1: 96.00%**

Bold values indicate the best-performing result in each column.

### 4.6 Overall quantitative comparison

A consolidated quantitative comparison was performed to examine whether the proposed architecture maintains competitive performance across both image-level classification and pixel-level lesion segmentation. Evaluating these tasks together is important because high classification accuracy does not necessarily indicate accurate localization of symptomatic tissue, while strong segmentation alone may not guarantee reliable disease recognition. The comparison therefore includes representative convolutional, attention-based, instance-segmentation, and Transformer-enhanced architectures evaluated using task-specific metrics. Classification performance is characterized by accuracy, precision, recall, and F1-score, whereas segmentation is assessed using Dice, IoU, precision, and foreground recall. Presenting these measures separately provides a more transparent view of model behavior and prevents performance in one task from compensating for deficiencies in the other. The architecture-matched comparison also evaluates whether the joint learning strategy can preserve discriminative image-level information while improving spatial delineation of affected regions. Consequently, the analysis provides an integrated assessment of predictive reliability, lesion localization capability, and the balance achieved between the two complementary objectives within the proposed CuFormer U-Net framework under consistent experimental conditions.

**Figure 7** reproduces the canonical values from **Tables 2**, **3**. The classification panel compares the five strongest relevant classifiers and reports 96.7% accuracy for CuFormer U-Net. The segmentation panel compares the five strongest dense-prediction models and reports 92.4% mean image-wise Dice for CuFormer U-Net. Error bars are intentionally omitted because independent-run variability was not available; the chart is descriptive rather than inferential.

### 4.7 Ablation study

The archived component-wise ablation adds the Transformer bottleneck (TB), attention-guided skip connections (AGSC), disease-guided feature modulation (DGFM), boundary refinement (BAR), and deep supervision (DS) sequentially to the baseline. **Figure 9**; **Table 5** report the same fixed-split point estimates. The erroneous **Figure 9** label of 0.996 for the TB IoU has been corrected to 0.696. Because repeated-seed outputs and module-level profiling were unavailable, the ablation establishes descriptive incremental changes but not statistical significance or a verified cost-benefit ratio.

**Figure 9:**
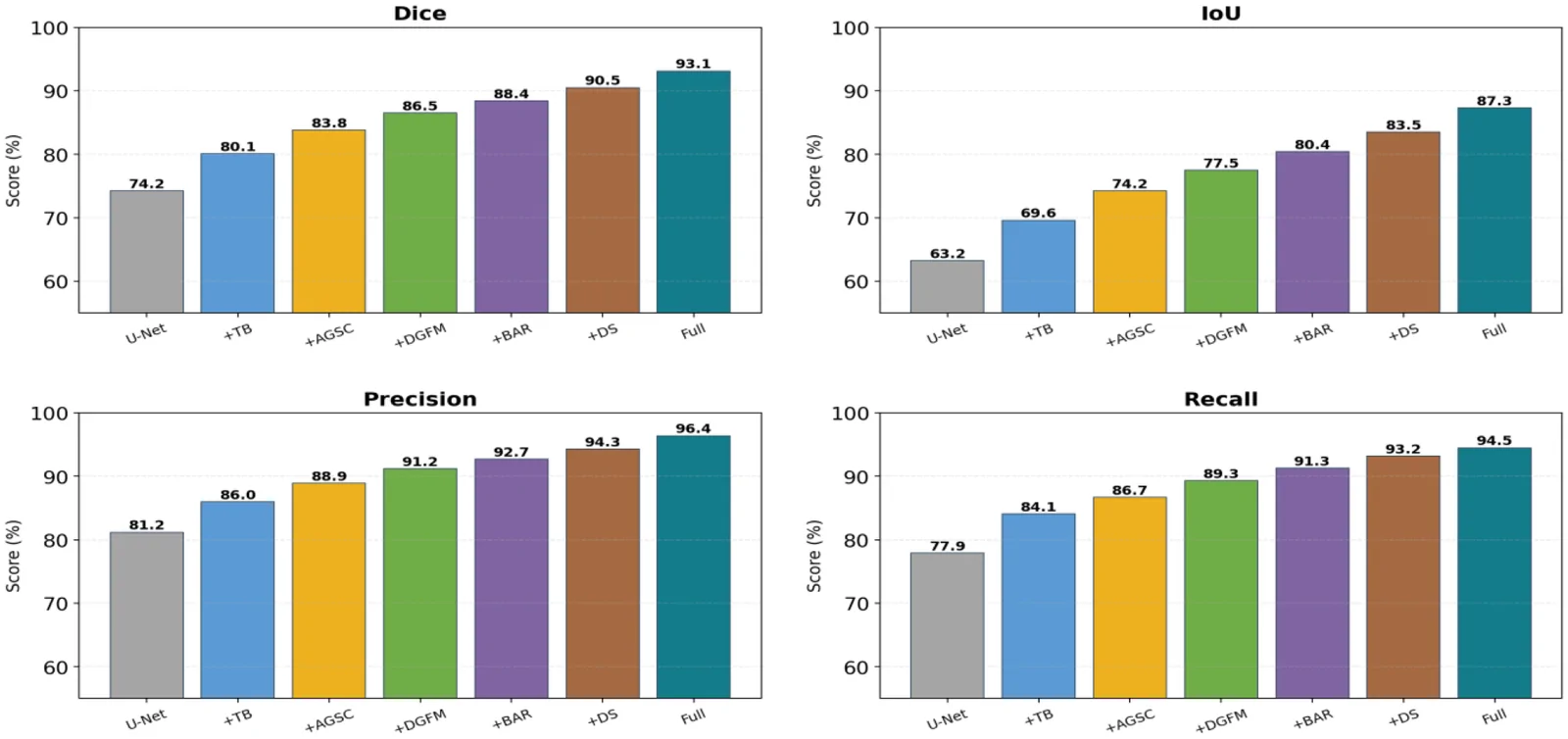
Corrected fixed-split component-wise ablation point estimates; the former TB IoU label of 0.996 is corrected to 0.696, and unverified complexity and variability panels are removed.

**Table 5:** Component-wise fixed-split ablation of CuFormer U-Net on the internal validation protocol.

Configuration	Dice (%)	IoU (%)	F1-score (%)	Precision (%)	Recall (%)
Baseline U-Net	74.2	63.2	79.5	81.2	77.9
+ Transformer bottleneck (TB)	80.1	69.6	85.0	86.0	84.1
+ Attention-guided skip connections (AGSC)	83.8	74.2	87.8	88.9	86.7
+ Disease-guided feature modulation (DGFM)	86.5	77.5	90.2	91.2	89.3
+ Boundary-aware refinement (BAR)	88.4	80.4	91.7	92.7	91.3
+ Deep supervision (DS)	90.5	83.5	93.5	94.3	93.2
Full model	**93.1**	**87.3**	**95.4**	**96.4**	**94.5**

The ablation values are sequential point estimates. Parameters, FLOPs, latency, memory, and repeated-run variability were not available in the archived record and are not fabricated. Bold values indicate the best-performing result in each column.

**Figure 9** demonstrates a consistent progressive improvement in segmentation performance as successive components are incorporated into the CuFormer U-Net architecture. The baseline U-Net achieved 74.2% Dice, 63.2% IoU, 81.2% precision, and 77.9% recall. Introducing the Transformer bottleneck (TB) increased these metrics to 80.1%, 69.6%, 86.0%, and 84.1%, respectively, indicating improved contextual representation. Attention-guided skip connections (AGSC) further raised Dice to 83.8%, while disease-guided feature modulation (DGFM) increased it to 86.5%. Subsequent boundary-aware refinement (BAR) and deep supervision (DS) produced additional gains, reaching 90.5% Dice and 83.5% IoU before full integration. The complete configuration achieved the highest performance, with 93.1% Dice, 87.3% IoU, 96.4% precision, and 94.5% recall. Overall, the monotonic improvements across all four metrics support the complementary contribution of the incorporated modules; however, these fixed-split point estimates should be validated through repeated paired experiments before statistical significance or causal contributions are inferred.

**Table 5** presents the component-wise fixed-split ablation results and demonstrates a progressive improvement in segmentation performance as additional components are incorporated into CuFormer U-Net. The baseline U-Net achieved 74.2% Dice, 63.2% IoU, 79.5% F1-score, 81.2% precision, and 77.9% recall. Adding the Transformer bottleneck increased Dice to 80.1% and IoU to 69.6%, followed by further improvements to 83.8% and 74.2%, respectively, after introducing attention-guided skip connections. Disease-guided feature modulation subsequently raised Dice to 86.5%, while boundary-aware refinement increased it to 88.4%. With deep supervision, Dice reached 90.5%, accompanied by 83.5% IoU, 93.5% F1-score, 94.3% precision, and 93.2% recall. The complete configuration produced the highest values across all metrics, achieving 93.1% Dice, 87.3% IoU, 95.4% F1-score, 96.4% precision, and 94.5% recall. Relative to the baseline, these values correspond to cumulative improvements of 18.9 percentage points in Dice and 24.1 points in IoU. The consistently increasing metrics indicate that the sequential architectural extensions are associated with improved lesion delineation. Nevertheless, these results represent descriptive cumulative differences from a fixed-split sequential ablation and should not be interpreted as independent causal contributions of individual modules without repeated-run or leave-one-component-out experiments.

## 5 Discussion

The fixed-split results are consistent with the hypothesis that structured interaction between classification and segmentation may be useful, but they do not establish statistical superiority. CuFormer U-Net exceeded CoAtNet-0 by 2.3 accuracy points and MedT by 0.5 Dice points in the recorded comparisons. The architecture does more than attach independent heads: classification probabilities form a disease embedding that modulates bottleneck features and conditions skip gates, while the shared encoder receives gradients from both tasks.

The Transformer bottleneck produced the largest early ablation gain. This result agrees with evidence that shifted-window Transformers improve practical cucumber recognition by modeling spatially separated symptoms (Wang et al., 2022). Full-resolution attention would be unnecessarily expensive, whereas 64 bottleneck tokens permit global interaction at moderate cost. The multi-scale context module prepares these tokens with dilated local evidence and global pooling, reducing the risk that patch embedding discards small spots.

Attention-guided skip connections improved segmentation by suppressing irrelevant encoder activations. UCTransNet similarly demonstrated that U-Net skip pathways should be treated as selective communication channels rather than unconditional concatenation (Wang et al., 2022b). The proposed gate extends that principle by incorporating disease embedding *d*, which helps distinguish true lesions from leaf veins, shadow boundaries, and background vegetation. Qualitative outputs in **Figure 8** confirm fewer fragmented false positives and better preservation of irregular symptom contours.

Disease-guided modulation improved both classification consistency and lesion localization. Multi-task Transformer research has shown that explicit cross-task interaction can outperform isolated decoders when tasks share spatial and semantic structure (Ye and Xu, 2022). The present design remains simpler than universal segmentation systems such as OneFormer because only two agricultural tasks are optimized (Jain et al., 2023). The boundary branch then addresses a different failure mode: interior pixels dominate conventional region losses, so uncertain edges receive insufficient gradient. Explicit boundary targets increased internal IoU from 77.5% to 80.4% at the corresponding ablation stage.

Qualitative examples include clutter, occlusion, variable lighting, dense spots, and edge necrosis, but they do not constitute a stratified robustness experiment. The manuscript therefore describes these cases as illustrative and does not claim demonstrated robustness to each factor. YOLO-Cucumber reported that deformable sampling and fine-resolution detection can benefit small targets in complex scenes (Liu et al., 2025), whereas dense masks additionally support affected-area estimation. Cross-study ranking remains inappropriate because augmentation, splitting, and task definitions differ (Shahid and Zohaib, 2025).

Computational complexity remains unresolved empirically. Restricting global attention to 64 bottleneck tokens is intended to reduce cost relative to full-resolution self-attention, but the available archive does not contain verified parameter counts, FLOPs, inference time, peak memory, or CPU results. Deployment feasibility therefore cannot yet be established. Profiling, pruning, distillation, and evaluation on the target mobile-robot hardware are required before making efficiency claims.

The practical implications extend beyond disease naming. Pixel masks permit severity scoring, longitudinal monitoring, and spatially selective spraying. Confidence-calibrated classification can triage uncertain images for expert review, while boundary maps can guide higher-resolution reinspection. The shared encoder also reduces duplicate computation compared with deploying separate classification and segmentation networks.

Several limitations materially constrain interpretation. The segmentation cohort contains only 400 images from a restricted greenhouse context, so rare phenotypes and environmental variation may be underrepresented and confidence intervals may be wide. Source-image splitting prevents derivative-image leakage, but plant, leaf-instance, location, and acquisition-session grouping could not be verified. Annotator identities, independent masks, disagreement logs, and inter-annotator agreement were not archived. The 100-image condition-shift cohort lacks recoverable quantitative outputs. Results are single-run point estimates without seed variability or significance tests, and computational profiling is incomplete. The public classification and greenhouse segmentation cohorts also use different taxonomies. These limitations preclude claims of statistical superiority, formal external validation, or deployment readiness.

## 6 Conclusion

This study developed CuFormer U-Net, a multi-task deep learning framework integrating local convolutional encoding, compact Transformer-based contextual modeling, classification-conditioned feature modulation, gated skip transfer, deep supervision, and boundary refinement for automated cucumber leaf analysis. On the three-class Kaggle benchmark, the proposed model achieved a fixed-split classification accuracy of 96.7%, together with a macro F1-score of 96.0%, demonstrating reliable discrimination among the evaluated cucumber leaf conditions. On the separate greenhouse binary visible-symptom segmentation task, CuFormer U-Net achieved a mean image-wise Dice score of 92.4%, macro IoU of 83.1%, foreground precision of 93.2%, and foreground recall of 91.7%, indicating effective localization of symptomatic tissue. Furthermore, the complete configuration in the sequential ablation experiment reached 93.1% Dice and 87.3% IoU on the internal validation protocol. Although the results demonstrate promising classification and segmentation performance, they remain descriptive and should be interpreted cautiously. Future work should include independent-seed experiments, paired statistical testing, comprehensive annotation provenance, and quantitative external validation across diverse datasets. Reproducible computational profiling and prospective evaluation under real greenhouse conditions are also required to establish statistical reliability, robustness, generalizability, and practical deployment readiness.

## Data Availability

The original contributions presented in the study are included in the article/supplementary material. Further inquiries can be directed to the corresponding author.
